# Characteristics of multiple early gastric cancer and gastric high-grade intraepithelial neoplasia

**DOI:** 10.1097/MD.0000000000036439

**Published:** 2023-12-08

**Authors:** Yudai Chen, Liping He, Xiaoling Zheng

**Affiliations:** a Shengli Clinical Medical College of Fujian Medical University, Fuzhou, China; b Department of Digestive Endoscopy, Fujian Provincial Hospital South Branch, Fuzhou, China; c Department of Digestive Endoscopy, Fujian Provincial Hospital, Fuzhou, China.

**Keywords:** endoscopic submucosal dissection, high-grade intraepithelial neoplasia, multiple early gastric cancer

## Abstract

This study evaluated the clinical characteristics of multiple early gastric cancer (MEGC) and high-grade intraepithelial neoplasia (HGIN) treated by endoscopic submucosal dissection. The clinical profiles of 23 patients with MEGC treated by endoscopic submucosal dissection from January 2008 to June 2019 at the Fujian Provincial Hospital or Fujian Provincial Hospital South Branch were analyzed. The following information was extracted from clinical records: general data, preoperative conditions, and pathological data of each lesion after surgery. In total, 23 patients with MEGC or HGIN were evaluated (average age 64 ± 6 years, 17 (73.9%) males). MEGC and HGIN accounted for 4.9 percent of all cases, in which 19 (4.1%) were synchronous multiple cancers and 4 (0.8%) cases were metachronous multiple cancers. Lesions of synchronous and metachronous MEGC groups did not differ in age, sex, smoking history, alcohol consumption, family history of tumors, *Helicobacter pylori* infection, mucosal background atrophy, or intestinal metaplasia (*P* > .05). The vertical locations of primary and secondary lesions of MEGC were correlated (*R* = 0.395, *P* = .034). The primary and secondary lesions of MEGC shared the same macroscopic subtype (*R* = 0.590, *P* = .015), infiltration depth (*R* = 0.455, *P* = .014), and pathological subtype (*R* = 0.736, *P* < .001). MEGC and HGIN were located in close proximity. Pathologic types tended to be low-grade malignancies. The macroscopic type, histology type, and infiltration depth of the 2 lesions were significantly correlated. When detecting early gastric cancer, we should inspect the stomach and carefully consider the pathological characteristics, to improve the diagnosis of MEGC.

## 1. Introduction

Gastric cancer is a malignant tumor with high incidence and mortality. According to statistics from 2018, the incidence of gastric cancer in the world is among the top 5 among all tumors, and the mortality rate is within the top 3 of all tumors.^[1]^ Recently, with the development of digestive endoscopy technology, the detection rate of early gastric cancer (EGC) has continued to increase. EGC generally refers to gastric cancer that is limited to the gastric mucosa or submucosa, regardless of whether there is lymph node metastasis.^[2]^ The prognosis of EGC is good. If there is no evidence of lymph node metastasis, the 5-year survival rate can be >90%.^[3]^ Endoscopic submucosal dissection (ESD) is currently the main treatment option for EGC. However, some researchers have identified multiple occurrences of postoperative gastric cancer histopathology.^[4]^ Despite the detection rates of multiple EGC (MEGC) and high-grade intraepithelial neoplasia (HGIN) have been gradually increasing in recent years, and MEGC has also received increased attention. Currently, there is a scarcity of research on gastric cancer, with a predominant focus on synchronous gastric cancer (SMEGC). The patient population is mainly comprised of individuals who have undergone surgical procedures,^[5]^ Kim et al^[6]^ analyzed the independent risk factors for simultaneous multiple cancers to be male gender and submucosal invasion. Although Lee et al^[7]^ analyzed the characteristics of MEGC, the conclusions drawn were limited. There is a lack of research on the endoscopic findings of MEGC and post-ESD outcomes. The existing evidence from studies is insufficient. Therefore, it is important to summarize the characteristics of MEGC lesions and explore the key features that require attention during screening and review of EGC.

## 2. Methods

### 2.1. Study population

Information relative to hospitalized patients with MEGC who underwent 1 or more ESD procedures at the Fujian Provincial Hospital or Fujian Provincial Hospital South Branch from January 2008 to June 2019 and pathologically confirmed as EGC or HGIN after surgery were included. Age, sex, medical history, morphology, location, and pathological data of endoscopic lesions were collected.

### 2.2. Definition of terms

*MEGC*^[8]^: Refers to EGC with more than two independent EGC lesions in the stomach. According to the Moertel standard, MEGC should meet the following criteria: (1) each lesion is a cancerous lesion confirmed by pathology; (2) normal gastric mucosa intervals interspersed among different lesions are visible by microscopy; and (3) lesions resulting from local infiltration or metastasis are not included.

*Synchronous multiple EGC*^[8]^: An EGC present in more than 2 lesions simultaneously or new lesions identified within 12 months of the original finding.

*Metachronous multiple EGC (MMEGC*)^[8]^: Referred to the EGC in which new EGC lesions are found outside the primary location 12 months after the resection of the primary lesion.

Definition of primary lesion and secondary lesion: (1) in metachronous multiple EGC, the earlier lesion was defined as the primary lesion. (2) In SMEGC, when multiple lesions present the same infiltration depth, the larger lesion was considered to be the main lesion, when multiple lesions presented inconsistent depths of infiltration, the deepest lesion was considered the main lesion, with 3 or greater simultaneous lesions, the second major lesion was considered to be the secondary lesion.^[9]^ In pathology staging, the infiltration of tumor cells into the mucosal layer and mucosal muscle layer both fall under stage T1a, and there is no lesion infiltration into the submucosal layer (T1b) in this study. We primarily classifies the primary SMEGC lesions based on lesion size.

### 2.3. Classification standards

**Figure 1.**
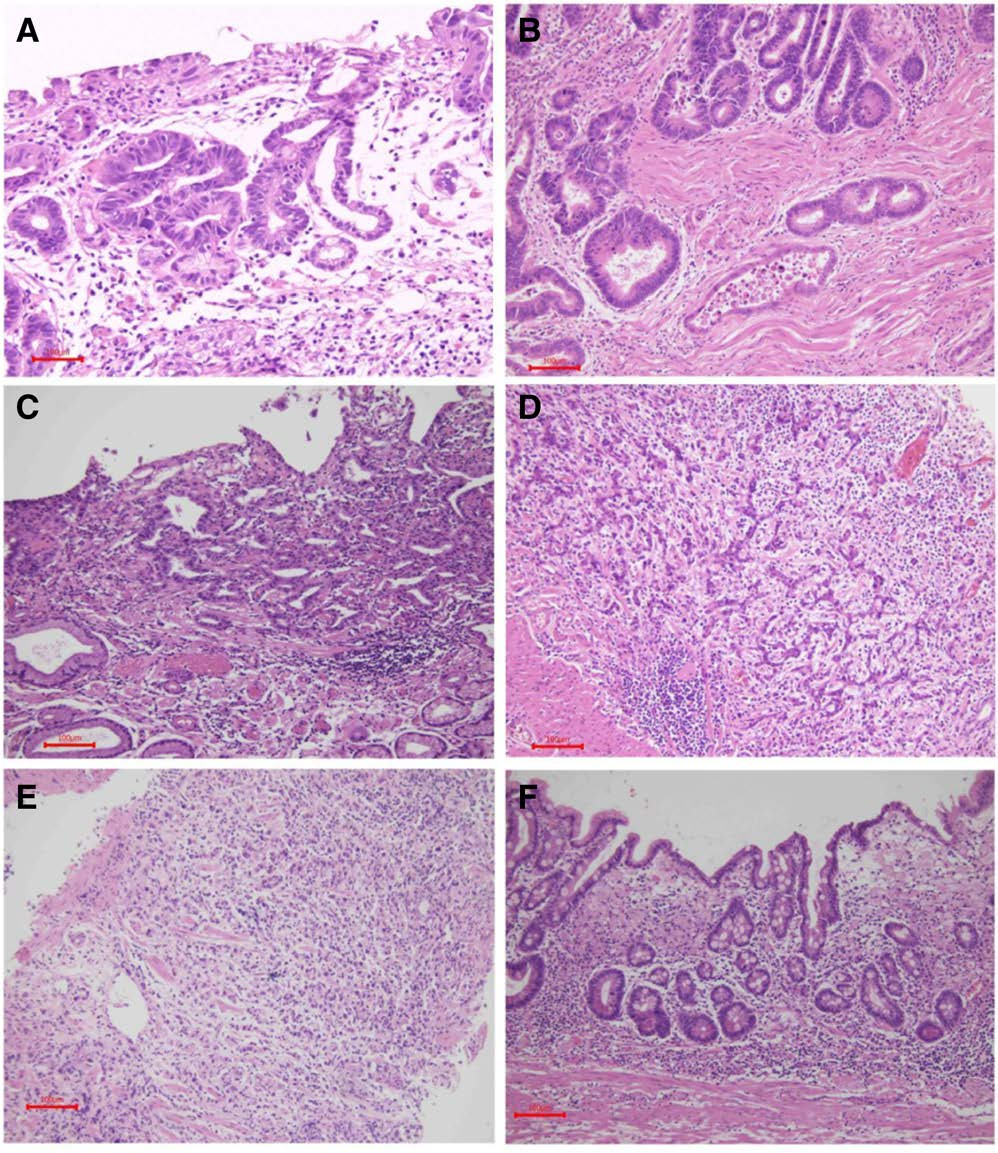
The pathological classification of early gastric cancer (H&E × 100). (A) High grade intraepithelial neoplasia (HGIN). Differentiated adenocarcinoma, (B) was well differentiated tubular adenocarcinoma, (C) was moderately differentiated tubular adenocarcinoma. Undifferentiated adenocarcinoma, (D) was poorly differentiated adenocarcinoma, (E) was low adherent adenocarcinoma, (F) was signet ring cell carcinoma.

In accordance with the Paris classification,^[10]^ the morphological classification of EGC was classified into 3 groups based on microscopic evaluation: polypoid (type 0-I), flat elevated (type 0-IIa, 0-IIb, 0-IIa + IIc), and superficial depressed (type 0-IIc, 0-III). According to the World Health Organization (WHO) classification criteria, the pathological classification of EGC^[11]^ was divided into 3 groups: HGIN, differentiated adenocarcinoma, undifferentiated adenocarcinoma (Fig. 1). Based on the horizontal distribution in the stomach EGC was divided into: anterior wall, middle curvature, posterior wall, and major curvature.^[9,11]^ Tumor location (long axis) was classified by dividing the stomach into three equal sections: upper (cardia, fundus, and upper body), middle (midbody, lower body, and angle), and lower (antrum and prepylorus).

### 2.4. Surgical procedure and pathological diagnosis

For endoscopic treatment and pathologic diagnosis of EGC, preoperative blood examination and abdominal computed tomography scan were performed to further eliminate co-morbidities and to obtain informed consent from the patient’s family. All ESD experts have treated at least 100 patients and have at least 2 years of ESD experience. Magnifying chromoendoscopy was used to assess the extent of the lesion, endoscopic morphological typing of gastric cancer, and endoscopic ultrasonography were used to assess the depth of lesion infiltration. Olympusq260j endoscope with feeding water pipe, D-201-11804 transparent cap, Dual knife, electric hemostatic forceps, injection needle for one-time endoscope, rotatable repeated opening, and closing soft tissue clamp, electrode plate, rubber plate, insect needle, CO_2_ air pump, and other accessories were used for the procedure.

During the surgical procedure, the boundary of the lesion was marked and the injection needle was used to perform multi-point submucosal injections. After the lesion was fully lifted, part of the mucosa surrounding the lesion was dissected along the pre-marked area along the edge of the lesion, followed by a deeper dissection into the submucosa to achieve a one-time complete resection of the entire lesion. Based on the patient’s condition, APC, hot biopsy forceps, hemostatic clamps, and other techniques are used to treat the resection wounds. If perforation was suspected, titanium clamps were used for clamping.^[12]^ After the ESD, patients were asked to fast with water deprivation for 2 to 3 days, and intravenous application of proton pump inhibitors was started on the same day. Postoperative specimens were stretched with insect needles, fixed on foam plates, and fixed in 8% formalin for 24 hours before embedding. After embedding, specimens were sectioned every 2 mm and stained with hematoxylin-eosin, and the specimens were observed under light microscopy.

### 2.5. Statistical analysis

Statistical analysis was performed using SPSS 23.0 software. Normally distributed data are expressed as mean ± standard deviation (x ± SD). Count data are expressed in frequency and percentage (%). Fisher precision test was used for inter-group comparison and the rank sum test was used for non-normal and uneven variance data. Column Correlation Analysis was used to analyze correlations. *P* < .05 was considered statistically significant.

### 2.6. Ethical approval and consent to participate

The study was conducted in accordance with the Declaration of Helsinki and approved by the Biomedical Ethics Committee of Shengli Clinical Medical College of Fujian Medical University. The ethics committee approval number is K2019-03-089. As this was a retrospective study, all patients gave up informed consent.

## 3. Results

### 3.1. Clinical features of patients with MEGC and HGIN

From January 2008 to June 2019, a total of 469 patients were pathologically confirmed with EGC or HGIN following ESD surgery. Of these, only 23 patients with MEGC or HGIN were included in the study. All patients had a review deadline of June 2019. MEGC and HGIN accounted for 4.9% (23/469) of all EGC cases, including 19 cases (4.1%) of SMEGC and 4 cases (0.8%) of MMEGC. There were 17 (73.9%) male patients with MEGC, with a mean age of 64 ± 6 years. There were no statistically significant differences in age, sex, smoking, alcohol consumption, or family histories, *Helicobacter pylori* infection, or in background mucosal atrophy and intestinal metaplasia (*P* > .05). The details are summarized in Table 1.

**Table 1:** Clinical features of patients with MEGC and HGIN.

Factors(n)	SMEGC (n = 19)	MMEGC (n = 4)	*P* value
*Sex*			
Male	13 (68.4%)	4 (100%)	.539
Female	6 (31.6%)	0 (0%)	
*Age*			
<60	3 (15.8%)	2 (50%)	.209
60–70	13 (68.4%)	1 (25%)	
≥70	3 (15.8%)	1 (25%)	
*Smoking history*			
Yes	5 (26.3%)	1 (25.0%)	.999
No	14 (73.7%)	3 (75.0%)	
*Alcohol consumption history*			
Yes	3 (15.8%)	0 (0%)	.999
No	16 (84.2%)	4 (100%)	
*Family history*			
Yes	4 (21.1%)	2 (50%)	.270
No	15 (78.9%)	2 (50%)	
*Helicobacter pylori infection*			
Yes	15 (78.9%)	2 (50%)	.270
No	4 (21.1%)	2 (50%)	
*Background mucosal atrophy*			
Mild	1 (5.3%)	1 (25%)	.075
Moderate	11 (57.9%)	0 (0%)	
Severe	7 (36.8%)	3 (75%)	
*Background mucosal intestinal metaplasia*			
Mild	1 (5.3%)	1 (25%)	.387
Moderate	17 (89.5%)	2 (50%)	
Severe	1 (5.3%)	1 (25%)	

*Note*: *P* value < .05 was significant.

MMEGC = metachronous multiple early gastric cancer; SMEGC = synchronous multiple early gastric cancer.

### 3.2. Correlation analysis of the distribution of primary and secondary lesions

When the primary lesion was located in the upper third of the stomach, 16.7% of the secondary foci were also located in the upper third of the stomach. When the primary lesion was in the middle third of the stomach, 37.5% of the secondary lesions were located in the same area. When the primary lesion was in the lower third of the stomach, 33.3% of the secondary foci were located in the same region. A total of 30.4% of the MEGC cases exhibited the same vertical distribution of primary and secondary foci, and there was a significant correlation between primary and secondary lesions and vertical distribution (*R* = 0.395, *P* = .034, Table 2). Detailed data on the distribution of vertical locations of SMEGC and MMEGC lesions are described in Table S1-1 (Supplemental Digital Content, http://links.lww.com/MD/K952, which illustrates the distribution of vertical locations of SMEGC and MMEGC lesions).

**Table 2:** Correlation analysis of the vertical distribution of primary and secondary lesions.

Primary lesions	Secondary lesions			Total	*r*-value	*P*-value
Vertical distribution	Upper	Middle	Lower		0.395	.034
Upper	1 (16.7%)	3 (50.0%)	2 (33.3%)	6 (26.1%)		
Middle	3 (37.5%)	3 (37.5%)	2 (25.0%)	8 (34.8%)		
Lower	0 (0.0%)	6 (66.7%)	3 (33.3%)	9 (39.1%)		
Total	4 (17.4%)	12 (52.2%)	7 (30.4%)	23 (100.0%)		

*Notes*: *R value* was analyzed using the Column Correlation analysis. *P* value < .05 was significant.

From the perspective of horizontal distribution, no major curvature lesions were identified in this study. Overall, 56.5% of the primary and secondary lesions were distributed in the same horizontal position. The primary and secondary lesions in patients with MEGC showed no significant correlation with the horizontal distribution (*R* = 0.506, *P* = .095, Table 3). There were 11 cases (57.9%) and 2 cases (50%) of SMEGC and MMEGC respectively distributed in the same regions (Table S1-2, Supplemental Digital Content, http://links.lww.com/MD/K953, which illustrates the distribution of horizontal locations of SMEGC and MMEGC lesions).

**Table 3:** Correlation analysis of the horizontal distribution of primary and secondary lesions.

Primary lesions	Secondary lesions			Total	*r*-value	*P*-value
Horizontal distribution	Anterior wall	Posterior wall	Middle curvature		0.506	.095
Anterior wall	4 (36.4%)	6 (54.5%)	1 (9.1%)	11 (47.8%)		
Posterior wall	1 (11.1%)	7 (77.8%)	1 (11.1%)	9 (39.1%)		
Middle curvature	0 (0.0%)	1 (33.3%)	2 (66.7%)	3 (13.1%)		
Total	5 (21.7%)	14 (60.9%)	4 (17.4%)	23 (100.0%)		

Notes: *R value* was analyzed using the Column Correlation analysis. *P* value < .05 was significant.

### 3.3. Correlation of primary and secondary lesions of MEGC with their histological pathology

Morphologically, when the primary lesion was of the polypoid type, approximately 66.7% of the secondary lesions were also of the polypoid type. When the primary lesion was the flat elevated type, approximately 70.0% of the secondary lesions had a similar type. Approximately 60.0% of the secondary lesions were superficially depressed when the primary lesion was superficially depressed. Further, 65.2% of the primary and secondary lesions shared the same morphology under visual microscopy. There was a statistically significant correlation between the primary and secondary foci and their microscopic morphology (*R* = 0.590, *P* = .015, Table 4).

**Table 4:** The correlation between the primary and secondary lesions of MEGC and morphology.

Primary lesions	Secondary lesions			Total	*r*-value	*P*-value
Morphology type	Polypoid	Flat elevated	Superficially depressed			
Polypoid	2 (66.7%)	1 (33.3%)	0 (0.0%)	3 (13.1%)	0.59	.015
Flat elevated	0 (0.0%)	7 (70.0%)	3 (30.0%)	10 (43.5%)		
Superficially depressed	1 (10.0%)	3 (30.0%)	6 (60.0%)	10 (43.5%)		
Total	3 (13.1%)	11 (47.8%)	9 (39.1%)	23 (100.0%)		

Notes: *R value* was analyzed using the Column Correlation analysis. *P value* < 0.05 was significant.

Twelve cases (63.2%) in the SMEGC and 3 cases (75%) in the MMEGC presented identical primary and secondary lesion microscopic morphology (Table S2-1, Supplemental Digital Content, http://links.lww.com/MD/K954, which illustrates the morphology type of SMEGC and MMEGC lesions). In addition, in terms of infiltration depth, 82.6% of the secondary lesions presented the same depth of infiltration as the primary lesions. There was a correlation between the depth of infiltration of the primary and secondary lesions (*R* = 0.455, *P* = .014, Table 5). Detailed data for SMEGC and MMEGC lesions are shown in Table S2-2 (Supplemental Digital Content, http://links.lww.com/MD/K955, which illustrates the infiltration depth of SMEGC and MMEGC lesions). In terms of the postoperative pathological type, 65.2% of cases shared the same pathological type for both primary and secondary lesions, with a significant correlation between primary and secondary lesions (*R* = 0.736, *P* < .001, Table 6, Table S2-3, Supplemental Digital Content, http://links.lww.com/MD/K956, which illustrates the pathologic type of SMEGC and MMEGC lesions).

**Table 5:** The correlation between the primary and secondary lesions of MEGC and infiltration depth.

Primary lesions	Secondary lesions		Total	*r*-value	*P*-value
Infiltration depth	Mucosal layer	Mucosal muscle layer			
Mucosal layer	16 (84.2%)	3 (15.8%)	19 (82.6%)	0.455	.014
Mucosal muscle layer	1 (25.0)	3 (75.0%)	4 (17.4%)		
Total	17 (73.9%)	6 (26.1%)	23 (100.0%)		

*Notes*: *R value* was analyzed using the Column Correlation analysis. *P* value < .05 was significant.

**Table 6:** The correlation between the primary and secondary lesions of MEGC and pathology.

Primary lesions	Secondary lesions			Total	*r*-value	*P*-value
Pathologic type	HGIN	Differentiated	Undifferentiated			
HGIN	10 (55.6%)	8 (44.4%)	0 (0%)	18 (78.3%)	0.736	<.001
Differentiated	0 (0%)	4 (100%)	0 (0%)	4 (17.4%)		
Undifferentiated	0 (0%)	0 (0%)	1 (100%)	1 (4.3%)		
Total	10 (43.5%)	12 (52.2%)	1 (4.3%)	23 (100%)		

*Notes*: *R* value was analyzed using the column correlation analysis. *P* value < .05 was significant.

HGIN = high-grade intraepithelial neoplasia.

## 4. Discussion

In recent years, a growing number of EGC cases have been identified during screening for EGC in China. The number of cases of EGC treated by endoscopy is rapidly increasing. ESD is a recognized as a minimally invasive surgical procedure for EGC, but the incidence of SMEGC or MMEGC after surgery is greater than that following traditional surgical resection. The subsequent occurrence of synchronous or metachronous tumors in locations other than the primary lesion is one of the main concerns of this emerging surgical procedure^[6]^ and is likely due to the fact that ESD surgery preserves more gastric mucosa than traditional surgery.^[13]^

Statistically, SMEGC accounts for 9% to 14% of all cases of gastric cancer, and MMEGC accounts for 5.1% to 14% of all cases of EGC after ESD.^[7]^ In our study, SMEGC and MMEGC accounted for 4.1% and 0.8% of all EGC lesions, respectively, a difference that may be due to the Japanese pathology standards used in previous studies in which more patients were diagnosed with EGC, than the WHO standards used in the present study. This difference may also be related to the more stringent inclusion criteria of our study. We excluded cases of submucosal infiltration, vascular infiltration, lymph node metastasis, and positive margins to distinguish SMEGC, MMEGC, and early local recurrence as much as possible to improve the reliability of our results. Previous studies^[14,15]^ have indicated that the incidence of MEGC in patients with *Helicobacter pylori* eradication after ESD was 0.8% to 4.1%. In this study, routine eradication of *Helicobacter pylori* after ESD surgery significantly reduced the incidence of MMEGC, which may be one of the reasons for the lower rate of MEGC.

The current detection rate of EGC in China is about 10%, and has reached approximately 30% in some areas in recent years, which is nonetheless lower than in South Korea and Japan.^[16]^ In addition to the different pathological standards used for diagnosis, the following factors should also be considered: China has not systematically and extensively carried out an EGC screening program; technical skills among endoscopists are not equally distributed, and the knowledge of EGC is limited; population compliance is poor, and a good follow-up system has not be established.^[17]^ Therefore, exploring how to improve the detection rate of EGC and MEGC in China is our main task at present.

The currently available reports of MEGC in China are mainly case reports. With the deepening of the understanding of EGC, we predict that the detection rate of MEGC will increase further.^[18,19]^ Our findings showed that MEGC was more prevalent among males, older patients, and among individuals with *Helicobacter pylori* infection, gastric mucosal atrophy, and intestinal metaplasia, which suggests that the above may be considered risk factors for MEGC. Additional studies are required to confirm the impact of these potential risk factors during follow-up. In addition, most of the multiple cases of EGC in this study were identified within 1 year, and thus, may have been missed during the initial endoscopic screening. This suggests the importance of closer follow-ups within 1 year after the first operation. Nonetheless, metachronous lesions were detected 3 years after the endoscopic resection, suggesting that the long-term follow-up after ESD should not be overlooked.^[20]^

This study also found similarities among morphological features of the MEGC lesions, which were more often of the flat type, which is consistent with the results of previous studies.^[21]^ The morphology of the MEGC lesions was similar under direct microscopy (*R* = 0.590, *P* = .015). In terms of lesion distribution characteristics, over 70% of the lesions identified were located in the middle and lower thirds of the stomach, and there was a significant correlation between the primary and vertical distribution of these lesions (*R* = 0.359, *P* = .034). These results are in accordance with previous studies describing multiple gastric cancers,^[22]^ whereby multiple MEGC lesions occurred in proximity of each other.

Kim et al^[3]^ reported the similarities in focal microscopic classification, with most of the SMEGC foci also distributed in the distal stomach, with a significant correspondence between the horizontal and vertical spatial distribution of the primary and secondary foci. The “tumor collision” hypothesis explains the above phenomenon. It is speculated that progressive gastric cancer is formed by the fusion of multiple foci of SMEGC, thus the location and morphology of the foci will be similar. This suggests that during the follow-up of EGC screening, we should focus on the proximity of the tumor and pay closer attention to foci presenting the same morphology, to reduce the possibility of missing SMEGC lesions. A greater number of lesions were identified in the distal stomach in this study, which was not in accordance with the study by Kitamura et al,^[21]^ In hence, smaller SMEGC lesions seemed more likely to appear as flat typed lesions in the middle and upper stomach. Thus, when an EGC lesion was found during post-ESD follow-up, in addition to the careful observation of the proximal location of the lesion, the height of the gastric body should also be carefully monitored to identify flat lesions to avoid missed diagnoses.

The pathologic type of this study was predominantly HGIN and differentiated carcinoma tended to be less malignant, a result consistent with previous studies.^[23]^ There was a strong agreement between primary and secondary lesions in terms of pathology type and depth of infiltration. These results agree with a related study,^[3]^ which proposed the hypothesis of “field carcinogenesis,” based on the proposed common carcinogenic background present throughout the gastric mucosa.

For the same individual, conditions such as *Helicobacter pylori* infection, genetic and environmental factors, and exposure to carcinogens act on the entire gastric mucosa, and thus may result in similar tumor pathology and invasive behavior. This hypothesis explains the similarity in location, morphology, and pathological characteristics between SMEGC and MMEGC observed in this study. In addition, risk factors such as *Helicobacter pylori* infection, atrophic gastritis, and intestinal chemosis acted mainly on the distal stomach, suggesting this was a more tumor-prone region in the stomach. Furthermore, most of the multiple carcinomas detected in this study occurred in the distal stomach, which supports the hypothesis of “field carcinogenesis.”

This study has certain limitations. The detection of Helicobacter pylori infection in some patients may be affected by medication, leading to the possibility of false negatives, which should be confirmed through prospective research. Given the rarity of multiple gastric cancers, the sample size in this study is relatively small, and further expansion of the sample size is needed in future studies

In conclusion, with the increasing detection rate of MEGC, the presence of SMEGC and MMEGC are not as rare as suggested by previous reports. Paying closer attention to high-risk groups, combining morphology, distribution, and pathology of MEGC with careful and closer screening and follow-up, will improve the detection and cure rates of MEGC.

## Acknowledgments

We thank the contributions of the staff of the Department of Digestive Endoscopy of Fujian Provincial Hospital and Fujian Provincial Hospital South Branch in Fuzhou, China. Thanks to Dr Liping He for reviewing the research methods in this study, and the methods and techniques mentioned are appropriate for the research. The authors are accountable for all aspects of the work in ensuring that questions related to the accuracy or integrity of any part of the work are appropriately investigated and resolved.

## Author contributions

**Conceptualization:** Liping He, Xiaoling Zheng.

**Data curation:** Yudai Chen.

**Formal analysis:** Yudai Chen.

**Funding acquisition:** Xiaoling Zheng.

**Investigation:** Yudai Chen.

**Methodology:** Yudai Chen.

**Project administration:** Yudai Chen.

**Resources:** Yudai Chen.

**Software:** Yudai Chen.

**Supervision:** Liping He, Xiaoling Zheng.

**Validation:** Liping He, Xiaoling Zheng.

**Visualization:** Liping He, Xiaoling Zheng.

**Writing – original draft:** Yudai Chen.

**Writing – review & editing:** Liping He, Xiaoling Zheng.

## Supplementary Material










