# Supplementary material for: Characteristics of multiple early gastric cancer and gastric high-grade intraepithelial neoplasia
Source: Medicine (Baltimore). 2023 Dec 8;102(49):e36439. doi: 10.1097/MD.0000000000036439 (PMC10713190; doi:10.1097/MD.0000000000036439)
Supplement: Supplementary file 1 [file medi-102-e36439-s001.docx]

**Supplementary Table S1-1** Distribution of vertical locations of SMEGC and MMEGC lesions.

| Type of MEGC | Primary lesions | Secondary lesions | | | Total | Similar location |
| --- | --- | --- | --- | --- | --- | --- |
|  | Vertical distribution | upper | middle | lower |  |  |
| SMEGC(n=19) | upper | 1 | 2 | 2 | 5 | 1 |
|  | middle | 1 | 3 | 2 | 6 | 3 |
|  | lower | 0 | 5 | 3 | 8 | 3 |
|  | Total | 2 | 10 | 7 | 19 | 7(36.8%) |
| MMEGC(n=4) | upper | 0 | 1 | 0 | 1 | 0 |
|  | middle | 2 | 0 | 0 | 2 | 0 |
|  | lower | 0 | 1 | 0 | 1 | 0 |
|  | Total | 2 | 2 | 0 | 4 | 0(0%) |

Notes: SMEGC, Synchronous multiple early gastric cancer. MMEGC, Metachronous multiple early gastric cancer.
