# Supplementary material for: Characteristics of multiple early gastric cancer and gastric high-grade intraepithelial neoplasia
Source: Medicine (Baltimore). 2023 Dec 8;102(49):e36439. doi: 10.1097/MD.0000000000036439 (PMC10713190; doi:10.1097/MD.0000000000036439)
Supplement: Supplementary file 3 [file medi-102-e36439-s003.docx]

**Supplementary Table S2-1** Morphology type of SMEGC and MMEGC lesions.

| Type of MEGC | Primary lesions | Secondary lesions | | | Total | Similar location |
| --- | --- | --- | --- | --- | --- | --- |
|  | Morphology type | polypoid | flat elevated | superficially depressed |  |  |
| SMEGC(n=19) | polypoid | 2 | 1 | 0 | 3 | 2 |
|  | flat elevated | 0 | 6 | 3 | 9 | 6 |
|  | superficially depressed | 1 | 2 | 4 | 7 | 4 |
|  | Total | 3 | 9 | 7 | 19 | 12(63.2%) |
| MMEGC(n=4) | polypoid | 0 | 0 | 0 | 0 | 0 |
|  | flat elevated | 0 | 1 | 0 | 1 | 1 |
|  | superficially depressed | 0 | 1 | 2 | 3 | 2 |
|  | Total | 2 | 1 | 1 | 4 | 3(75%) |

Notes: SMEGC, Synchronous multiple early gastric cancer. MMEGC, Metachronous multiple early gastric cancer.
