# Supplementary material for: Characteristics of multiple early gastric cancer and gastric high-grade intraepithelial neoplasia
Source: Medicine (Baltimore). 2023 Dec 8;102(49):e36439. doi: 10.1097/MD.0000000000036439 (PMC10713190; doi:10.1097/MD.0000000000036439)
Supplement: Supplementary file 4 [file medi-102-e36439-s004.docx]

**Supplementary Table S2-2** Infiltration depth of SMEGC and MMEGC lesions.

| Type of MEGC | Primary lesions | Secondary lesions | | Total | Similar location |
| --- | --- | --- | --- | --- | --- |
|  | Infiltration depth | Mucosal Layer | Mucosal muscle layer |  |  |
| SMEGC(n=19) | Mucosal Layer | 13 | 3 | 16 | 13 |
|  | Mucosal muscle layer | 1 | 2 | 3 | 2 |
|  | Total | 14 | 5 | 19 | 15(78.9%) |
| MMEGC(n=4) | Mucosal Layer | 3 | 0 | 3 | 3 |
|  | Mucosal muscle layer | 0 | 1 | 1 | 1 |
|  | Total | 4 | 0 | 4 | 4(100%) |

Notes: SMEGC, Synchronous multiple early gastric cancer. MMEGC, Metachronous multiple early gastric cancer.
