# Supplementary material for: Characteristics of multiple early gastric cancer and gastric high-grade intraepithelial neoplasia
Source: Medicine (Baltimore). 2023 Dec 8;102(49):e36439. doi: 10.1097/MD.0000000000036439 (PMC10713190; doi:10.1097/MD.0000000000036439)
Supplement: Supplementary file 5 [file medi-102-e36439-s005.docx]

**Supplementary Table S2-3** Pathologic type of SMEGC and MMEGC lesions.

| Type of MEGC | Primary lesions | Secondary lesions | | | Total | Similar location |
| --- | --- | --- | --- | --- | --- | --- |
|  | Pathologic type | HGIN | Differentiated | Undifferentiated |  |  |
| SMEGC(n=19) | HGIN | 9 | 6 | 0 | 15 | 9 |
|  | Differentiated | 0 | 4 | 0 | 4 | 4 |
|  | Undifferentiated | 0 | 0 | 0 | 0 | 0 |
|  | Total | 9 | 10 | 0 | 19 | 13(68.4%) |
| MMEGC(n=4) | HGIN | 1 | 2 | 0 | 3 | 1 |
|  | Differentiated | 0 | 0 | 0 | 0 | 0 |
|  | Undifferentiated | 0 | 0 | 1 | 1 | 1 |
|  | Total | 2 | 1 | 1 | 4 | 2(50%) |

Notes: SMEGC, Synchronous multiple early gastric cancer. MMEGC, Metachronous multiple early gastric cancer.
